# Central Park

**Published:** 1861-04

**Authors:** 


### HALL'S JOURNAL OF HEALTH.

Our Legitimate Scope is almost boundless: for whatever begets pleasurable and harmless feelings, promotes Health; and whatever induces disagreeable sensations, engenders Disease.

WE AIM TO SHOW HOW DISEASE MAY BE AVOIDED, AND THAT IT IS BEST, WHEN SICKNESS COMES, TO TAKE NO MEDICINE WITHOUT CONSULTING A PHYSICIAN.

Vol. VIII.] APRIL, 1861. [No. 4.

### CENTRAL PARK.

THE Central Park of New-York City contains within the boundaries of Fifty-ninth and One hundred and tenth streets and Fifth and Eighth avenues, eight hundred and forty-four (844) acres. The land cost five million seven hundred thousand dollars. Two million six hundred thousand dollars have been expended on its construction to January first, eighteen hundred and sixty-one. The contemplated additional expenditure is two millions of dollars. It will cost the interest of one hundred and fifty thousand dollars to keep it in order. The entire cost of the Central Park will, therefore, be very near ten and a half millions of dollars, or about twelve thousand five hundred dollars an acre. It was planned by Olmstead and Vaux, the former, Frederic Law Olmstead, being Superintendent.

RECAPITULATION.

| | |
|---|---|
| Total contents, . . . . . . . . . | Acres 844 |
| Gross cost of land, . . . . . . . . | $5,700,000 |
| Expended in construction to Jan. 1st, 1861, . | 2,600,000 |
| Additional expenditures contemplated, . . . | 2,000,000 |
| Annual cost of maintenance, interest on, . . | 150,000 |
| Total cost, . . . . . . | $10,450,000 |

The Central Park is two and a half miles long and half a mile wide. From the Battery to the nearest entrance is four miles and three quarters, Miles 4¾

The lake at summer level is 7 feet deep and contains Acres 20

" winter level is 4 " for skating, 18

| | |
|---|---|
| The old Reservoir covers, acres, . . . . . | 36 |
| The new " " " . . . . . . | 106 |
| Average number of hands on Park for 1860, per day, | 3000 |
| Largest number on any one day, . . . . . | 3666 |

The Central Park in winter is used for driving, sleighing, and skating; no more enlivening sight can be presented, than that of thousands of men and women, boys and girls, in variegated dress and costume, sporting themselves in every direction, while hundreds of carriages are passing and repassing, or are stationary on the banks of the lake, their occupants looking on with interest and delight, in proportion to the nearness of their connection with the skaters.

In summer, the Park is not less inviting to riders on horseback or in carriages, to promenaders and pedestrians of every grade. It will be the resort of the lover; of nurses with children; of friend with friend. The old, the invalid, and the sad will go there to get a breath of pure air, to see the grass, the green leaves, and the flowers, and in melancholy musings to think of days departed; while the young and healthy and happy-hearted will find their feelings in unison with all that is there to exhilarate and to gladden.

It is to be hoped that row-boats will be placed on the lake, at a moderate charge by the hour, to give an agreeable and healthful exercise to all who need it, especially to the young, as a means of developing the chest and bringing out their muscular activity.

There will be a five-mile foot-path for the lounger, the lover, and the contemplative; a nineteen-mile carriage-drive over an easy graded and beautifully smooth turnpike, and a separate bridle-road of twenty miles, now up, now down, straight on, then winding around some projecting rock or miniature mountain, thus giving all invaluable opportunities for perfecting themselves in the most exhilarating and health-giving of the graces, riding on horseback.

Arrangements are made by which persons may reach the Park in the city cars for five cents, and when there, saddle-horses can be had by the hour, thus giving to citizens at a moderate cost, the opportunity of daily horseback-riding, without the expense of keeping a horse, or the discomfort of having to ride for miles over rough and dusty and crowded streets before the enjoyment of the country commences.

It would be an admirable arrangement, if the school children of the city could be turned out on the Park, every afternoon, to spend an hour or two in their plays and pastimes. They leave school at three in the afternoon; by four o'clock they are through with their dinners, and on an average, could reach the Park at four and a half, to remain an hour and a half or two hours. The healthful influences of such an arrangement would be incalculable. But from these chances of invigorating the constitution and laying up a stock of health for years to come, they are remorselessly cut off, by the customs of all schools, public and private, of giving such lessons to be mastered from 3 P.M. until 9 next morning, as require the full interval; and yet with inconceivable stupidity, parents and editors contemplate this enormity with perfect indifference, or (and more likely) are too much engaged in making money or discussing politics to give the subject of the health of their children any efficient consideration.

PARKS OF NEW-YORK CITY.

| Name. | Value in 1856. | Acres. | Roods. | Poles. | Feet. |
|---|---|---|---|---|---|
| Battery,.................. | $3,000,000 | 10 | 2 | 22 | 239 |
| Bowling Green,........... | 135,000 | .. | 2 | 9 | 253 |
| City Hall Park,............ | 3,000,000 | 10 | 3 | 14 | .. |
| Duane,.................... | 15,000 | .. | .. | 21 | 66 |
| Five Points,............... | 15,000 | .. | .. | 24 | 193 |
| Hudson Square,........... | Private. | 4 | .. | 24 | 68 |
| Washington Square,....... | 816,000 | 9 | 2 | 39 | 246 |
| Tompkins Square,......... | 337,000 | 10 | 2 | 1 | 112 |
| Abingdon Square,.......... | 12,000 | .. | .. | 33 | 36 |
| Union Square,............. | 504,000 | 3 | 1 | 34 | 253 |
| Stuyvesant Square,....... | 196,000 | 3 | 3 | 28 | 217 |
| Gramercy Park,........... | Private. | 1 | 2 | 30 | 92 |
| Madison Square,........... | 520,000 | 6 | 3 | 19 | 47 |
| Bloomingdale Square,..... | | 18 | .. | 9 | 36 |
| Hamilton Square,.......... | 97,000 | 15 | .. | .. | .. |
| Observatory Place,......... | | 25 | 3 | 2 | 160 |
| Manhattan Square.,........ | 88,000 | 19 | .. | 8 | 182 |
| Mount Morris,............. | 40,000 | 20 | .. | 27 | 114 |
| | $8,775,000 | 161 | | | |
| Central Park,............. | 5,700,000 | 844 | | | |
| Total,................ | $14,475,000 | 1005 | | | |

PARKS OF THE WORLD.

| | Acres. |
|---|---|
| London: | |
| Green Park, | 56 |
| St. James', | 87 |
| Kensington, | 227 |
| Little Park, | 300 |
| Regent's, | 372 |
| Hyde Park, | 380 |
| Richmond Park, | 2250 |
| Windsor Park, | 3500 |
| Total Parks in London, | 6172 |
| Magdeburg Park and Garden, | 128 |
| Liverpool, Birkenhead, | 180 |
| Berlin, Thiergarten, | 200 |
| Munich, Englischer Park and Garden, | 500 |
| Baltimore, Maryland, | 539 |
| Vienna, Prater, | 1500 |
| Dublin, Phœnix, | 2000 |
| Paris: | |
| Bois de Boulogne, | 2158 |
| Versailles Garden, | 3000 |

